# Diagnostic performance of a multi-shell DTI protocol and its subsets with B-matrix spatial distribution correction in differentiating early multiple sclerosis patients from healthy controls

**DOI:** 10.3389/fneur.2025.1618582

**Published:** 2025-07-28

**Authors:** Artur Tadeusz Krzyzak, Julia Lasek, Agnieszka Slowik

**Affiliations:** ^1^AGH University of Krakow, Kraków, Poland; ^2^The LaTiS NMR - Tomography and Spectroscopy Laboratory, Kraków, Poland; ^3^Department of Neurology, Jagiellonian University Medical College, University Hospital in Krakow, Krakow, Poland

**Keywords:** diffusion tensor imaging, BSD-DTI, spatial systematic errors, multiple sclerosis, clinical trail protocol

## Abstract

**Introduction:**

This study investigates whether a multi-shell diffusion tensor imaging (DTI) protocol and its subsets can reliably distinguish healthy controls (HC) from patients with multiple sclerosis (MS) presenting with low Expanded Disability Status Scale (EDSS) scores and mild MRI findings.

**Methods:**

To enhance accuracy, spatial systematic errors in diffusion measurements were corrected using the B-matrix Spatial Distribution method (BSD-DTI). We examined the discriminative potential of fractional anisotropy (FA) and mean diffusivity (MD) across three broad brain regions: whole brain (WB), white matter (WM), and gray matter (GM), using both the full protocol and its subsets. Additionally, we employed a more detailed classification strategy based on segmentation into 95 regions of interest (ROIs), analyzing FA, MD, axial diffusivity (AD), and radial diffusivity (RD) under a stringent statistical criterion.

**Results:**

While the protocol and each subset showed a comparable ability to differentiate between HC and MS groups, substantial variability in metric values across protocols highlights the limited utility of directly comparing DTI metrics between acquisition schemes.

**Discussion:**

The results emphasize the importance of accounting for spatial systematic errors when selecting optimal protocols for clinical and research applications.

## 1 Introduction

Diffusion Tensor Imaging (DTI) is an MRI technique that utilizes the phenomenon of water diffusion (1, 2) as a natural source of contrast. Since its introduction nearly three decades ago (3), DTI has become an important modality in neuroradiology, with increasing utility in the imaging of the musculoskeletal system, abdomen, and heart (4–8). A distinctive advantage of DTI lies in its ability to yield quantitative metrics derived from the eigenvalues and eigenvectors of the diffusion tensor, which are closely related to the underlying tissue microstructure (9–11).

Nevertheless, the comparability of DTI metrics across scanners, protocols, and sites remains limited. This is largely due to the dependence of the measured diffusion tensor on MRI hardware and sequence-specific parameters (12–15), resulting in an apparent rather than absolute tensor representation. Moreover, DTI is sensitive to random noise, affecting the signal-to-noise ratio (16, 17), as well as systematic spatial errors induced by gradient field inhomogeneities (18–23). While the former can be mitigated by increasing voxel size or signal averaging, the latter requires the precise characterization of the spatial error distribution specific to the scanner and protocol in use.

In this study, we aim to determine whether a multi-shell diffusion tensor imaging (DTI) protocol and its subsets can reliably distinguish healthy individuals from patients with multiple sclerosis (MS) with low Expanded Disability Status Scale (EDSS) scores, and to identify which variant offers the best diagnostic performance in individuals with mild neurological and magnetic resonance imaging (MRI) findings. To ensure accuracy, spatial systematic errors in diffusion measurements were corrected using the B-matrix Spatial Distribution method (BSD-DTI).

In contrast to previous work (31), which analyzed the discriminative power of DTI metrics in a different context, we evaluate the effectiveness of fractional anisotropy (FA) and mean diffusivity (MD) across three large brain regions, whole brain (WB), white matter (WM), and gray matter (GM) using both the full protocol and its subsets. Additionally, we propose a more detailed classification approach based on the segmentation of the brain into 95 regions of interest (ROIs) and the analysis of four DTI-derived metrics: FA, MD, axial diffusivity (AD), and radial diffusivity (RD), applying a stringent statistical criterion.

The influence of protocol design on group differentiation is assessed through a comparison of several protocol variants acquired on an MRI system optimized for high signal-to-noise ratio (SNR) and corrected for spatial systematic errors related to gradient nonuniformities, offering insights into optimal acquisition strategies for clinical application.

## 2 Theory and related work

The phenomenon of spatial heterogeneity in magnetic field gradients, observed in Diffusion Weighted Imaging (DWI) and DTI, was first addressed by researchers in 2003 (18). They attributed this effect to the nonlinearity of magnetic field gradients generated by gradient coils. This spatial heterogeneity is undesirable in DWI/DTI as it introduces systematic errors, negatively affecting the accuracy of diffusion measurements. To mitigate this issue, Bammer introduced a correction based on characteristics in the form of spherical harmonic functions that describe the magnetic field produced by the gradient coils. This correction led to significant improvements in the accuracy of DTI metric calculations (18).

An alternative approach to addressing this issue was proposed in 2008, utilizing diffusion tensor norms in the form of anisotropic and isotropic phantoms with well-defined structures and known distributions of diffusion tensors (24). Diffusion tensor norms here refer to the known, spatially resolved diffusion tensor field of the phantom for each voxel, serving as a reference diffusion tensor **D(r)**. By applying the Stejskal-Tanner (Equation 1), a system of equations is solved to accurately determine the spatial distribution of the **b(r)** matrix. This matrix represents the effective diffusion weighting at each voxel, reflecting local distortions caused by magnetic field gradient heterogeneity. The definition of **b(r)** is given explicitly in Equation 3. This approach does not require explicitly identifying the sources of heterogeneity in magnetic field gradients. Instead, the known diffusion tensor norms for each voxel within the studied region inside the RF coil are necessary. Methods for determining these reference diffusion tensor fields for isotropic and anisotropic phantoms have been described in detail in previous publications. This method, referred to as B-matrix Spatial Distribution in DTI (BSD-DTI), was thoroughly described both theoretically and experimentally, with successful implementation on a 9.4 T MRI system (22).

A complementary method involves mapping the spatial distribution of magnetic field gradients by directly measuring the magnetic field (21, 25). From this mapping, the spatial distribution of the b(r) matrix can be derived. This technique demonstrates the inherent heterogeneity in magnetic field gradients. However, it raises a critical concern regarding the accuracy of determining the true distribution of the b(r) matrix when utilizing MR sequences different from the one intended for the specific experimental setup.

It should be noted that the above experimental observations are contrary to the current theoretical approach, namely that the Stejskal-Tanner (S-T) equation assumes the constancy of the diffusion gradient vector in space. An attempt was made to find a solution (20). The derived Equation 2, the Generalized Stejskal-Tanner (GS-T) equation for non-uniform magnetic field gradients, allows for the analysis of any gradient distribution. In addition, the classical S-T equation is a unique solution of GS-T, assuming the constancy of gradient vector *G* in space.

In the classical approach, the value of matrix *b* is calculated for time *t = 2τ*, i.e., at the spin echo center of the dependence,

(1) $$\ln(\frac{A(2\tau )}{A(0)})=-b:D$$

where:

- A*(2τ), A(0)* are the signal intensities with and without diffusion gradients,
- *TE* – echo time, *τ = TE/2,*
- *b* – symmetric 3×3 matrix,
- *D* – symmetric diffusion tensor second rank
$$b(t)=\int_{0}^{t}k(t\prime )k{\left(t\prime \right)}^{T}\mathrm{dt}\prime$$

With the gradient vector G(t) = [Gx(t), Gy(t), Gz(t)]^T^, the following relation applies where

$$k(t,\prime )=\gamma \int_{0}^{t\prime }G(t\prime \prime )\mathrm{dt}\prime \prime -2H(t\prime -\tau )k(\tau ),\text{and}\;$$

$$k(\tau )=\gamma \int_{0}^{\tau }G(t\prime \prime )\mathrm{dt}\prime \prime$$

In fact, the diffusion gradient vector *G* has a non-uniform distribution in space dependent on the MR scanner, the sequence and its parameters. The solution of the Bloch-Torrey equation proposed in (20), with this assumption of the nonlinearity of the magnetic field, led to the known empirical form of the S-T equation, where matrix b has a spatial relationship *b(r),*

(2) $$\ln(\frac{A(2\tau )}{A(0)})=-b(r):D$$

The above equation is true, assuming a spin echo signal and symmetry of magnetic field gradients. Then the spatial distribution *b(r)* has the form,

(3) $$b(r)\underline{\underline{\mathrm{def}}}L(r)\underset{=b(t)}{\underset{︸}{\int_{0}^{2\tau }k(t)k^{T}(t)\mathrm{dt}L^{T}(r)}}$$

The *L(r)* tensor, which Bammer called the coil tensor, is now called the field correction tensor. It can also be interpreted as the Jacobian matrix for coordinates changing from Cartesian ones to those given by a curvilinear coordinate system, in which the diffusion gradient vector *G* is constant in space.

Over the last twenty years, researchers have continued to develop the three approaches mentioned above (18, 21, 23, 24, 26). However, no approach has so far provided a definitive solution to the problem. Recently, a large clinical study performed on a modern 3 T MR scanner confirmed the existence of systematic errors related to the spatial heterogeneity of magnetic field gradients.

## 3 Materials and methods

### 3.1 Participants

**Figure 1:**
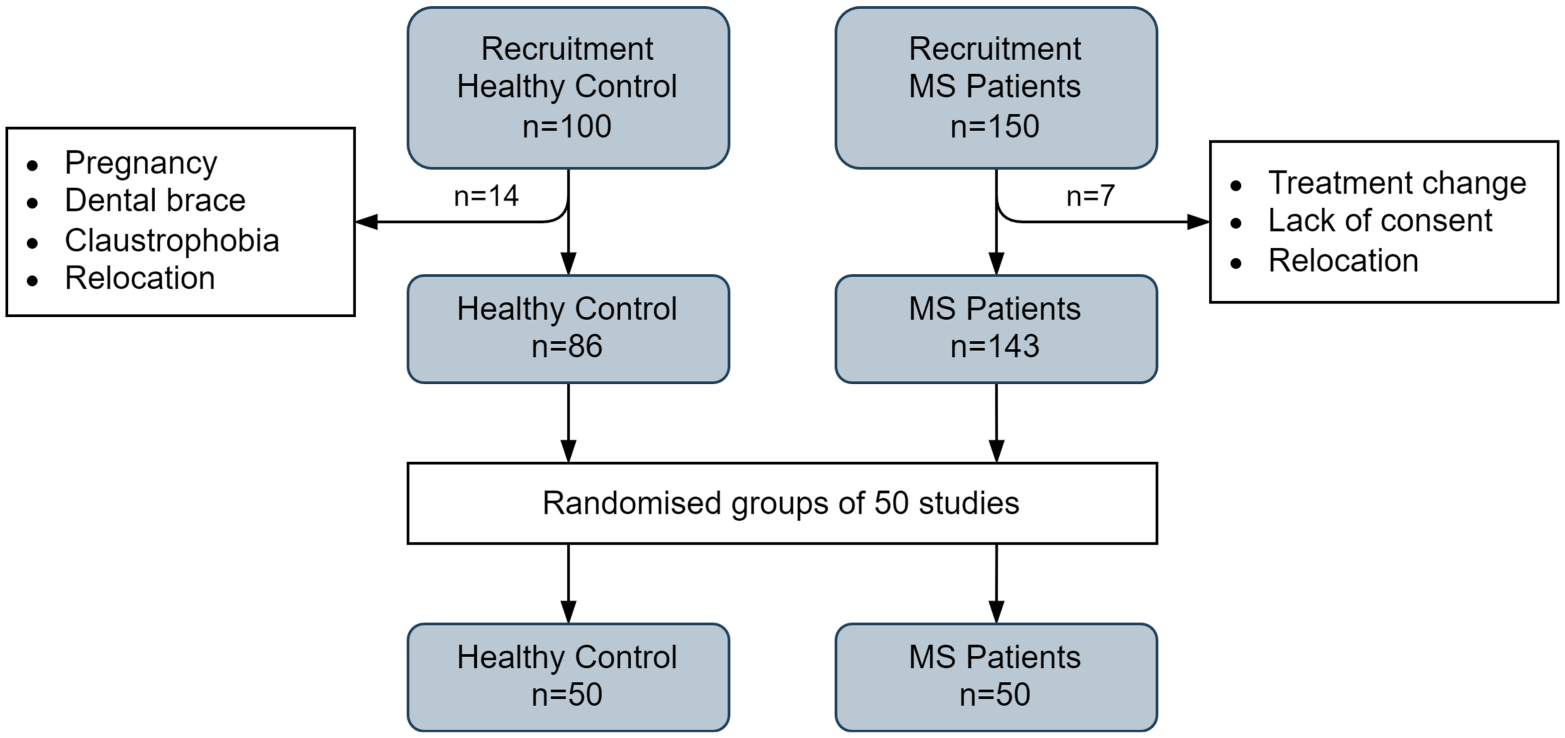
The flow chart shows the procedure for recruiting multiple sclerosis patients and healthy volunteers for MRI examinations.

Demographic characteristics are presented in Table 1, and the recruitment process for both patient and control groups is illustrated in Figure 1. The clinical study received a favorable opinion from the Bioethics Committee of the Regional Medical Chamber (Opinion No. 282/KBL/OIL/2020) on December 18, 2020.

**Table 1:** Demographic table.

Characteristic	HC	MS	*p*
Number of subjects	50	50	-
Male/female	16/34	15/35	1.0
Mean age (SD)	34.73 (7.80)	35.28 (6.95)	0.84
Median age (IQR)	34 (27–42)	35.5 (30–40)	0.84
Median EDSS (IQR)	-	1 (1–1.5)	-

HC, healthy control; MS, multiple sclerosis; EDSS, expanded disability status scale; IQR, interquartile ranges. Age of participants in years.

Participants were recruited at the University Hospital between January 2021 and January 2022. The multiple sclerosis (MS) group comprised individuals aged 18–50 years, diagnosed with relapsing–remitting MS, and presenting an Expanded Disability Status Scale (EDSS) score between 0 and 6.5.

MS patients were selected as they frequently present subtle, spatially diffuse microstructural brain changes that are particularly suitable for evaluating the effectiveness of technical corrections in DTI imaging.

Exclusion criteria included contraindications to MRI and pregnancy.

The healthy control (HC) group consisted of volunteers recruited from hospital staff and their family members. Eligible individuals were aged 18–50 years and had no clinical signs or symptoms indicative of MS. Volunteers with demyelinating lesions on MRI suggestive of multiple sclerosis or radiologically isolated syndrome were excluded.

Initially, 150 MS patients and 100 HC participants were enrolled. MRI data acquisition continued through August 2022. After exclusions, the final sample comprised 143 MS patients and 86 HC volunteers. For further analysis, a random subset of 50 participants from each group was selected. Details of the selection procedure are shown in Figure 1.

### 3.2 MRI protocol

Images were obtained using a 3 T Magnetom Vida fit scanner (Siemens, Germany) at the University Hospital, using the following imaging protocol: (a) T1-weighted magnetization-prepared rapid gradient echo (MPRAGE): repetition time (TR), 2,300 ms; echo time (TE), 2.99 ms; acquisition time (AT), 238 s; field of view (FOV), 242×250 mm^2^; voxel size, 0.98×0.98×1.2 mm^3^; (b) multi-shell DTI using spin-echo echo-planar imaging (SE-EPI): number of diffusion gradient directions (NDGD), NDGD = 20; *b*-value = 0, 1000, 2000 s/mm^2^; TR/TE = 3900/88 ms; AT = 680 s; FOV = 191×191 mm^2^; voxel size = 2.5×2.5×2.5 mm^3^. This primary DTI protocol, denoted as 1000/2000(40), was divided into subsets: (a) 1000(20): *b* = 0, 1000 s/mm^2^, NDGD = 20; (b) 1000(11): *b* = 0, 1000 s/mm^2^, NDGD = 11; (c) 1000(6): *b* = 0, 1000 s/mm^2^, NDGD = 6; (d) 2000(20): *b* = 0, 2000 s/mm^2^, NDGD = 20.

### 3.3 Study design

All participants underwent brain MRI examinations that included a diffusion tensor imaging (DTI) sequence as part of the protocol. For each participant, two identical measurements of an isotropic diffusion phantom were acquired using the same MRI scanner settings. The phantom and the participant’s head were positioned identically in the scanner’s laboratory coordinate system to ensure geometric consistency. Although phantom scans were typically performed immediately after the participant’s scan, the exact timing was not critical, as the systematic distortion remained stable throughout the four-year clinical study due to constant scanner hardware, fixed imaging protocol, and reproducible positioning. The first phantom acquisition was used to estimate the spatial distribution of the b(r) matrix corresponding to each diffusion gradient direction. The second, independent acquisition, with a different realization of Gaussian noise, was used to assess the effectiveness of the B-matrix Spatial Distribution (BSD) correction.

Phantom measurements were performed using a Siemens spherical phantom (D 165, solution C: 1.25 g NiSO₄ × 6 H₂O per 1000 g distilled H₂O; model 240, 10,496,625) and identical acquisition sequences and parameters to those used for human participants. Diffusion tensor metrics were then computed using both the conventional processing pipeline and an alternative approach incorporating systematic error correction via the BSD-DTI method.

### 3.4 Image analysis

#### 3.4.1 Region of interest (ROI) segmentation

The T1-weighted images were automatically segmented into anatomical structures using FastSurfer software (27). Since the segmentations of the MS brains contained some misclassifications, especially in the demyelinated areas, each was verified and manually corrected. FastSurfer provides whole-brain segmentation into 95 classes (95 ROIs) that were grouped in our study into 3 ROIs: whole brain (WB), white matter (WM), and gray matter (GM).

Two experienced data analysts performed all manual annotations, verified by two neurology and neuroradiology experts.

Subsequently, the ROIs were subjected to a resampling process to match the resolution of the DWI data. This resampling was done using the ResampleImageFilter from the SimpleITK library (28). The DWI images were designated as the reference for resampling, thereby defining the target resolution, spatial orientation, and dimensions. A nearest-neighbor interpolator was employed to calculate the intensity values of the resampled segmentations. Once resampled, the aligned segmentations were used to calculate DTI metrics within the specified regions.

#### 3.4.2 Calculation of DTI metrics

The diffusion tensor elements were calculated by minimizing the chi-square distribution for a system of 40, 20, 11 or 6 equations, depending on the subset version. Calculations were performed for each voxel in two cases: standard (STD), using a single b-matrix for each DWI and BSD, utilizing the b(r) distribution, offering a separate b-matrix for each voxel (22). The following metrics were calculated from the tensor components: FA–fractional anisotropy, MD–mean diffusivity, AD – axial diffusivity, RD – radial diffusivity.

Calculations and analyses were performed for each brain measurement and the twin phantom measurement for the same ROIs segmented from the T1 weighted brain image. An identical protocol was used in the measurements, and care was taken to ensure the identical location of the tested objects; the patient’s head and the phantom were in the laboratory reference system of the MR scanner.

The application of BSD correction algorithms, developed in C/C++ and compiled with Visual Studio 13.00 (Microsoft, USA), is available at: https://nmrlab.pl/en/bsd/.

#### 3.4.3 Correction of spatial systematic errors

The determination of the actual distribution of b(r) related to the spatial heterogeneity of magnetic field gradients was generally performed according to the principles of BSD-DTI (20, 22), with a new approach to calculating the spatial distribution b(r) of the matrix b for each DWI recently used in a clinical study spanning several years (29). The impact of systematic errors and the effectiveness of BSD correction were assessed by analyzing FA and MD distributions for 100 measurements and a single isotropic phantom measurement in ROIs identified from the corresponding brain imaging.

The influence of systematic errors and the effectiveness of BSD correction were assessed by analyzing the FA and MD distributions for 100 measurements (Phantoms; 50 each for HC and MS) and a single (1Phantom) measurement of an isotropic phantom in regions of interest identified from the corresponding brain imaging according to the rules given in 3.3 Study design.

The obtained FA for an isotropic phantom should ideally be 0, and its deviation is due to the influence of noise and systematic errors. Ideally, the MD for such a phantom should have a constant value and a standard deviation of 0.

#### 3.4.4 Statistical analysis

Statistical analyses were performed using the SciPy library (version 1.12). Normality of the data was first assessed with the Shapiro–Wilk test. As the distributions of key variables deviated from normality, non-parametric tests were applied in subsequent analyses.

The Mann–Whitney U-test was used to compare the HC and MS groups for variables such as age and diffusion tensor imaging (DTI) metrics. The Chi-square test assessed differences in categorical variables, such as sex distribution.

To evaluate the impact of BSD correction on DTI measurements, the Wilcoxon signed-rank test was used to compare standard (STD) and BSD-corrected values within subjects.

Effect sizes were also calculated to estimate the magnitude of observed differences. Effect size was interpreted according to Cohen’s thresholds: values above 0.3 were considered medium, and those above 0.5 were classified as large.

To control for false discovery due to multiple comparisons, *p*-values were adjusted using the Benjamini–Hochberg false discovery rate (FDR) correction method.

No covariates were added to the model, as the HC and MS groups were demographically well matched.

#### 3.4.5 A method of assessing the effectiveness of distinguishing between HC and MS groups

In addition to the results demonstrating the ability of DTI metrics—FA and MD—to differentiate between HC and MS groups in large ROIs (WB, WM, GM), we propose a more comprehensive method. This approach involves the analysis of four DTI metrics (FA, MD, AD, RD) across 95 ROIs, applying a stringent criterion for detecting group differences.

The criterion was met when at least one of the four metrics revealed a statistically significant difference (SSD) between HC and MS in each ROI (*p* < 0.005). A value of 100% indicates that SSDs were found in all 95 ROIs. As an additional evaluation, we report effect sizes, considering medium (> 0.3) and large (> 0.5) thresholds.

## 4 Results

### 4.1 Participants characteristics

Statistical analysis regarding age distribution revealed no statistically significant differences (SSD) between the two groups. The mean age in years and standard deviation (SD) for the MS group were 35.28 (SD = 6.95), and for the HC group were 34.73 (SD = 7.80), yielding a *p*-value of 0.84. Furthermore, the median ages in years and interquartile ranges (IQR) were established at 35.5 (IQR = 30–40) for the MS group and 34 (IQR = 27–42) for the HC group, with a corresponding p-value of 0.84.

### 4.2 Diffusion tensor metrics of the phantom measurements

#### 4.2.1 Estimation of noise and systematic errors

The FA and MD measures obtained for the isotropic phantom using the standard approach show the scale of the overall noise and systematic errors (Table 2). The results indicate that the worst accuracy of a single FA measurement (1Phantom) concerns subset 1,000(6); the highest FA = 0.0482 due to MD is subset 2000(20); the highest SD = 6.34E- 05. In turn, the best accuracy for both metrics is provided by subset 1,000(20): lowest FA = 0.0353 and lowest SD = 3.95E-05 for MD.

**Table 2:** Diffusion tensor metrics (FA and MD) obtained from 100 phantom measurements (Phantoms; 50 MS + 50 HC) and a single measurement (1Phantom), using the standard approach (STD) and after correction for spatial systematic errors with the BSD method.

B val (dirs)	Mean diffusivity		Fractional anisotropy	
	Phantoms (SD)	1Phantom (SD)	Phantoms (SD)	1Phantom (SD)
1000/2000 (40)				
STD	2.04E-03 (4.38E-05)	2.07E-03 (5.60E-05)	4.08E-02 (3.23E-03)	4.02E-02 (1.68E-02)
BSD	2.03E-03 (4.76E-05)	2.07E-03 (1.66E-05)	2.68E-02 (3.06E-03)	2.83E-02 (1.34E-02)
*p*	<0.001 (0.83)	<0.001 (0.23)	<0.001 (0.87)	<0.001 (0.76)
2000 (20)				
STD	2.04E-03 (4.26E-05)	2.07E-03 (6.34E-05)	4.51E-02 (3.29E-03)	4.54E-02 (1.94E-02)
BSD	2.03E-03 (4.71E-05)	2.07E-03 (2.10E-05)	3.36E-02 (3.65E-03)	3.55E-02 (1.70E-02)
*p*	<0.001 (0.65)	<0.001 (0.19)	<0.001 (0.87)	<0.001 (0.69)
1000 (20)				
STD	2.05E-03 (4.92E-05)	2.09E-03 (3.95E-05)	3.77E-02 (6.29E-03)	3.53E-02 (1.69E-02)
BSD	2.03E-03 (4.97E-05)	2.07E-03 (7.75E-06)	1.02E-02 (3.94E-03)	9.61E-03 (6.12E-03)
*p*	<0.001 (0.87)	<0.001 (0.49)	<0.001 (0.87)	<0.001 (0.87)
1000 (11)				
STD	2.05E-03 (4.91E-05)	2.09E-03 (4.15E-05)	4.14E-02 (6.99E-03)	3.88E-02 (1.95E-02)
BSD	2.03E-03 (4.93E-05)	2.07E-03 (1.33E-05)	1.74E-02 (4.29E-03)	1.67E-02 (1.10E-02)
*p*	<0.001 (0.87)	<0.001 (0.52)	<0.001 (0.87)	<0.001 (0.86)
1000 (6)				
STD	2.05E-03 (4.99E-05)	2.09E-03 (4.97E-05)	5.08E-02 (8.87E-03)	4.82E-02 (2.99E-02)
BSD	2.04E-03 (5.04E-05)	2.08E-03 (2.06E-05)	2.86E-02 (5.22E-03)	2.83E-02 (1.95E-02)
*p*	<0.001 (0.87)	<0.001 (0.38)	<0.001 (0.87)	<0.001 (0.82)

Data are mean with standard deviation (SD) in parentheses.

STD, standard; BSD, B-matrix Spatial Distribution. Mean diffusivity is given in mm^2^/s. A p-value less than.05 indicates a statistically significant difference; the corresponding effect size is specified in parentheses alongside.

#### 4.2.2 Elimination of the impact of spatial systematic errors

Table 2 also shows that the BSD approach can remove the effects of systematic errors on DTI metrics for an individual protocol and their subsets. The subset 1,000(20) demonstrated the highest efficiency, with the lowest FA = 0.00961 and the lowest SD = 7.75E-06 for MD.

### 4.3 Diffusion tensor metrics of HC and MS groups and single measurement

#### 4.3.1 Whole brain region of interest

FA and MD metric values for 50-person HC and MS groups, depending on protocol or its subset type, are presented in Table 3. Moreover, the evolution of metric values depending on group size is tracked in Supplementary Table S1. The sensitivity of the metrics to distinguish between groups is shown in the context of SSD and the associated effect size. These results are analyzed for each approach, STD or BSD, separately and between them. Visualizations of the FA and MD distributions obtained by the basic protocol, 1,000/2000(40), for sample HC and MS measurement in 2D axial, sagittal and coronal views, as well as 3D histograms for the entire ROI, are presented in Supplementary Figure S1. Identical data sets for subsets 1,000(20) and 1,000(6) are included in Supplementary Figures S2, S3. These types of subsets were selected due to their additional features. The most outstanding efficiency of eliminating systematic errors occurred in the first case, and the smallest possible number of directions of the diffusion gradient vector was found in the second case.

**Table 3:** Diffusion tensor metrics (FA and MD) obtained from 50 healthy controls (Control) and 50 multiple sclerosis patients (Patients), calculated using the standard approach (STD) and after correction for spatial systematic errors using the BSD method.

B val (dirs)	Mean diffusivity			Fractional anisotropy		
	Control (SD)	Patients (SD)	*p*	Control (SD)	Patients (SD)	*p*
1000/2000 (40)						
STD	8.33E-04 (3.16E-05)	8.79E-04 (4.83E-05)	<0.001 (0.51)	2.15E-01 (7.97E-03)	2.07E-01 (9.97E-03)	<0.001 (0.41)
BSD	8.30E-04 (3.17E-05)	8.77E-04 (4.86E-05)	<0.001 (0.50)	2.15E-01 (7.92E-03)	2.07E-01 (9.73E-03)	<0.001 (0.42)
*p*	<0.001 (0.77)	<0.001 (0.69)		0.552 (0.10)	0.194 (0.20)	
2000 (20)						
STD	7.71E-04 (2.88E-05)	8.14E-04 (4.32E-05)	<0.001 (0.52)	2.30E-01 (7.45E-03)	2.21E-01 (9.11E-03)	<0.001 (0.47)
BSD	7.69E-04 (2.90E-05)	8.12E-04 (4.35E-05)	<0.001 (0.52)	2.30E-01 (7.32E-03)	2.21E-01 (8.86E-03)	<0.001 (0.49)
*p*	<0.001 (0.71)	<0.001 (0.64)		<0.001 (0.51)	<0.001 (0.51)	
1000 (20)						
STD	1.05E-03 (4.75E-05)	1.11E-03 (7.11E-05)	<0.001 (0.45)	2.39E-01 (8.25E-03)	2.30E-01 (9.85E-03)	<0.001 (0.46)
BSD	1.04E-03 (4.75E-05)	1.10E-03 (7.11E-05)	<0.001 (0.45)	2.40E-01 (8.23E-03)	2.30E-01 (9.88E-03)	<0.001 (0.45)
*p*	<0.001 (0.87)	<0.001 (0.87)		0.627 (0.08)	0.065 (0.28)	
1000 (11)						
STD	1.04E-03 (4.81E-05)	1.10E-03 (7.13E-05)	<0.001 (0.46)	2.77E-01 (9.87E-03)	2.65E-01 (1.11E-02)	<0.001 (0.50)
BSD	1.03E-03 (4.81E-05)	1.09E-03 (7.11E-05)	<0.001 (0.45)	2.78E-01 (9.76E-03)	2.65E-01 (1.12E-02)	<0.001 (0.51)
*p*	<0.001 (0.87)	<0.001 (0.87)		<0.001 (0.55)	<0.001 (0.68)	
1000 (6)						
STD	9.98E-04 (5.11E-05)	1.06E-03 (7.28E-05)	<0.001 (0.44)	3.14E-01 (9.51E-03)	2.99E-01 (1.08E-02)	<0.001 (0.63)
BSD	9.95E-04 (5.10E-05)	1.06E-03 (7.34E-05)	<0.001 (0.44)	3.14E-01 (9.42E-03)	2.99E-01 (1.09E-02)	<0.001 (0.62)
*p*	<0.001 (0.85)	<0.001 (0.77)		0.904 (0.02)	0.665 (0.07)	

Values refer to the Whole Brain (WB) region of interest.

Data are mean with standard deviation (SD) in parentheses.

STD, standard; BSD, B-matrix spatial distribution. Mean diffusivity is given in mm^2^/s. A p-value less than 0.05 indicates a statistically significant difference; the corresponding effect size is specified in parentheses alongside.

Supplementary Tables S2A,B show the results for the other ROIs, white matter and gray matter.

#### 4.3.2 White matter and gray matter regions of interest

Like the Whole Brain region, Supplementary Tables S2A,B show the values of FA and MD metrics in the White Matter and Gray Matter regions for the 50-person HC and MS groups depending on the protocol and its subset type.

#### 4.3.3 Effectiveness of DTI metrics to distinguish HC and MS groups with BSD impact assessment

Table 4 summarizes the effectiveness of distinguishing between HC and MS groups and includes an evaluation of the impact of BSD correction. The analytical approach, based on a relatively stringent criterion for assessing DTI metrics, is detailed in Section 3.4.5. The first column (‘ANY’) reports the percentage of ROIs in which a statistically significant difference (SSD; *p* < 0.005) between groups was observed for at least one DTI metric, accompanied by a medium effect size (> 0.3). The subsequent four columns present the corresponding results for each metric. The right side of the table presents an analogous analysis, applying a stricter threshold of a large effect size (> 0.5).

**Table 4:** Comparison of the effectiveness in differentiating between healthy controls (HC) and multiple sclerosis (MS) patients across 95 ROIs.

B val (dirs)	HC vs. MS									
	Medium effect size					Large effect size				
	ANY	MD	FA	AD	RD	ANY	MD	FA	AD	RD
1000/2000 (40)										
STD	48.4%	38.9%	4.2%	16.8%	35.8%	4.2%	3.2%	0.0%	3.2%	3.2%
BSD	48.4%	38.8%	6.3%	16.8%	38.9%	5.3%	3.2%	0.0%	4.2%	3.2%
2000 (20)										
STD	54.7%	46.3%	4.2%	16.8%	40.0%	4.2%	3.2%	0.0%	3.2%	3.2%
BSD	54.7%	45.3%	8.4%	16.8%	38.9%	4.2%	3.2%	0.0%	3.2%	3.2%
1000 (20)										
STD	32.6%	24.2%	8.4%	16.8%	30.5%	1.1%	1.1%	0.0%	1.1%	1.1%
BSD	34.7%	26.3%	11.6%	16.8%	32.6%	2.1%	1.1%	0.0%	1.1%	2.1%
1000 (11)										
STD	50.5%	25.3%	28.4%	16.8%	34.7%	5.3%	1.1%	3.2%	1.1%	2.1%
BSD	49.5%	29.5%	29.5%	16.8%	35.8%	6.3%	2.1%	4.2%	1.1%	2.1%
1000 (6)										
STD	62.1%	25.3%	47.4%	12.6%	42.1%	13.7%	1.1%	12.6%	1.1%	2.1%
BSD	63.2%	25.3%	50.5%	12.6%	42.1%	15.8%	1.1%	14.7%	1.1%	2.1%

The table presents the percentage of the 95 ROIs in which statistically significant differences (SSD; *p* < 0.005) between HC and MS groups were observed for four DTI metrics: FA (fractional anisotropy), MD (mean diffusivity), AD (axial diffusivity), and RD (radial diffusivity). The “ANY” column indicates the percentage of ROIs where at least one of the four metrics met the SSD criterion together with the specified effect size. The left section of the table corresponds to a medium effect size threshold (> 0.3), and the right section to a large effect size threshold (> 0.5). A value of 100% denotes that SSDs with the given effect size were observed in all 95 ROIs for the respective protocol subset.

This approach highlights the diagnostic potential of both the full protocol and its subsets, and it can serve as a foundation for developing effective classification models. Notably, the 1000(6) subset, despite utilizing the minimal number of diffusion gradient directions, demonstrates unexpectedly strong discriminatory power, particularly based on FA and RD. However, this subset is also the most susceptible to noise-related variability. In contrast, the full protocol (1000/2000(40)) shows higher sensitivity for MD and RD. Interestingly, RD exhibits comparable discriminatory performance across the full protocol and all its subsets.

## 5 Discussion and conclusion

The DTI metrics included in Table 2 show the impact of the MR protocol and its subsets on the accuracy of the measurement of a single diffusion tensor, here water in an isotropic phantom. The diversity of subsets and the use of the BSD method also allowed for a clear distinction between the influence of Gaussian noise and the physical phenomenon related to the inhomogeneity of magnetic field gradients, generating systematic errors with a specific spatial distribution. After operating the BSD calibration, the most beneficial improvement effects are observed for the 1000(20) subset. The MD standard deviation decreased more than 5 times, from 1.9 to 0.37%, and the FA value decreased approximately 3.7 times from 0.0353 to 0.0096. The improvement recorded for the primary protocol— 1000/2000(40) is significant, although less spectacular. We note an approximately 3.4-fold reduction in the standard deviation of MD from 2.71 to 0.8% and a 1.4-fold decrease in FA from 0.0402 to 0.0283. Therefore, the removable component of systematic errors in the total noise for the MD measurement is as much as 80 and 70%, respectively. This effect will similarly apply to any apparent diffusion tensor in a voxel for any examined object. This is well illustrated by a strong SSD, typically *p* < 0.001, and an associated huge effect size, an effect size > 0.8 between the STD and BSD approaches.

The subsequent results in Table 3, Supplementary Table S1, and Supplementary Figures S1–S3 help us understand the impact of non-uniform magnetic field gradients in combination with the type of MR protocol’s subset on DTI metrics.

The effectiveness of distinguishing 50-person HC and MS groups with MD and FA parameters in the Whole Brain ROI is similar regardless of the STD or BSD protocol’s subset and approach adopted. In Table 3, we observe strong SSD (*p* < 0.001) combined with a large effect size (~ 0.4–0.6) between the mean values of the DTI metrics. Considering the group size (Supplementary Table S1), SSD with p < 0.001 is achieved already for 30 persons regardless of the protocol subset. Surprisingly, as demonstrated in Table 4, applying metrics for 95 ROIs and the combined criteria, an SSD with *p* < 0.005 and an effect size greater than medium (0.3) or large (0.5), the most influential criterion is FA and subset 1000(6), i.e., with the minimum number of directions of the diffusion gradient vector, regardless of the size of the measurement group.

In turn, the numerical values of MD and FA are highly variable depending on the protocol’s subset and insignificant due to the group size. The extreme MD variability reaches ~ 40% and occurs between subsets 2000(20) and 1,000(20). In the case of FA, this variability is even greater and reaches a value of ~50% when comparing the results for main protocol 1000/2000(40) and subset 1,000(6), ~ 0.021 and ~ 0.031. At the same time, the BSD correction did not introduce any significant changes numerically. Note, however, that these values (and distributions) between approaches are characterized by a strong SSD with *p* < 0.001, combined with a huge effect size (effect size > 0.8). The small variability in metrics between the STD and BSD approaches is easier to understand by looking simultaneously at the extensive distributions of FA and MD (Figures S1-S3), which suggest the dominant influence of anatomical variation on mean FA and MD. Moreover, large ROIs can average the impact of systematic errors, which introduce much greater variation locally.

The above observations lead to several important conclusions. First, the primary correction of the MD and FA distributions is achieved by accounting for the actual spatial distribution of magnetic field gradients present during acquisition. As a result, increasing the number of signal averages (accumulations) has only a marginal effect in experiments with sufficiently high SNR. Crucially, this correction procedure is entirely independent of the imaged object; it depends solely on the MRI system, the sequence type, and specific acquisition parameters (20, 32).

Furthermore, comparing DTI-derived metrics across different acquisition protocols has limited interpretive value. These metrics should be evaluated within the context of a specific protocol, and only after verifying and correcting for the impact of spatially dependent systematic errors in each region of interest (ROI).

In addition, a recent study (30) demonstrated the effect of BSD correction on DTI metrics and tractography, while incorporating standard preprocessing steps commonly used in diffusion MRI studies. These steps included denoising (Local PCA), Gibbs oscillations correction, eddy current correction, motion correction, B1 inhomogeneity correction, and rigid body registration for geometric distortion correction. The findings confirmed that BSD maintains its effectiveness across these conditions, with denoising showing the most significant additional benefit. This reinforces the practical utility of BSD in clinical and research protocols aiming for higher anatomical precision.

## Data Availability

The original contributions presented in the study are included in the article/Supplementary material, further inquiries can be directed to the corresponding author.
