# Supplementary material for: Diagnostic performance of a multi-shell DTI protocol and its subsets with B-matrix spatial distribution correction in differentiating early multiple sclerosis patients from healthy controls
Source: Front Neurol. 2025 Jul 28;16:1618582. doi: 10.3389/fneur.2025.1618582 (PMC12340240; doi:10.3389/fneur.2025.1618582)
Supplement: Supplementary file 1 [file Data_Sheet_1.docx]

**Supplementary Materials**

**Tables**

**Tables S1**. The evolution of Diffusion tensor metrics from 50 healthy control measurements (CONTROL) and 50 multiple sclerosis patients (PATIENTS) in the standard (STD) approach and after eliminating systematic errors (BSD) for Whole Brain ROI, depending on group size.

**10**

| B val (dirs) | Mean Diffusivity | | | Fractional Anisotropy | | |
| --- | --- | --- | --- | --- | --- | --- |
|  | **CONTROL (SD)** | **PATIENTS (SD)** | **p** | **CONTROL (SD)** | **PATIENTS (SD)** | **p** |
| 1000/2000 (40) |  |  |  |  |  |  |
| STD | 8.27E-04 (1.31E-05) | 8.80E-04 (3.92E-05) | <0.001 (0.78) | 2.15E-01 (4.17E-03) | 2.06E-01 (8.59E-03) | <0.05 (0.54) |
| BSD | 8.25E-04 (1.50E-05) | 8.77E-04 (4.01E-05) | <0.005 (0.73) | 2.16E-01 (4.28E-03) | 2.06E-01 (8.28E-03) | <0.05 (0.56) |
| p | 0.093 (0.53) | <0.05 (0.79) |  | 0.203 (0.40) | 0.721 (0.11) |  |
| 2000 (20) |  |  |  |  |  |  |
| STD | 7.67E-04 (1.44E-05) | 8.14E-04 (3.23E-05) | <0.001 (0.78) | 2.30E-01 (4.75E-03) | 2.21E-01 (7.39E-03) | <0.05 (0.54) |
| BSD | 7.66E-04 (1.64E-05) | 8.11E-04 (3.30E-05) | <0.005 (0.71) | 2.30E-01 (4.69E-03) | 2.21E-01 (7.12E-03) | <0.05 (0.57) |
| p | 0.241 (0.37) | <0.05 (0.79) |  | <0.05 (0.73) | 0.386 (0.27) |  |
| 1000 (20) |  |  |  |  |  |  |
| STD | 1.04E-03 (1.61E-05) | 1.11E-03 (6.67E-05) | <0.005 (0.73) | 2.39E-01 (5.65E-03) | 2.30E-01 (7.15E-03) | <0.01 (0.59) |
| BSD | 1.03E-03 (1.64E-05) | 1.10E-03 (6.72E-05) | <0.005 (0.71) | 2.40E-01 (5.65E-03) | 2.30E-01 (7.00E-03) | <0.01 (0.63) |
| p | <0.01 (0.89) | <0.01 (0.89) |  | 0.093 (0.53) | 0.508 (0.21) |  |
| 1000 (11) |  |  |  |  |  |  |
| STD | 1.02E-03 (1.80E-05) | 1.10E-03 (6.59E-05) | <0.005 (0.71) | 2.75E-01 (8.07E-03) | 2.65E-01 (6.87E-03) | <0.05 (0.56) |
| BSD | 1.02E-03 (1.83E-05) | 1.09E-03 (6.61E-05) | <0.005 (0.71) | 2.76E-01 (7.92E-03) | 2.66E-01 (6.91E-03) | <0.05 (0.56) |
| p | <0.01 (0.89) | <0.01 (0.89) |  | <0.05 (0.79) | <0.01 (0.89) |  |
| 1000 (6) |  |  |  |  |  |  |
| STD | 9.88E-04 (2.16E-05) | 1.06E-03 (6.36E-05) | <0.005 (0.71) | 3.10E-01 (7.41E-03) | 2.99E-01 (6.44E-03) | <0.005 (0.64) |
| BSD | 9.85E-04 (2.29E-05) | 1.06E-03 (6.55E-05) | <0.005 (0.71) | 3.11E-01 (7.45E-03) | 2.99E-01 (5.96E-03) | <0.005 (0.64) |
| p | <0.05 (0.76) | 0.074 (0.56) |  | 0.093 (0.53) | 0.959 (0.02) |  |

**20**

| B val (dirs) | Mean Diffusivity | | | Fractional Anisotropy | | |
| --- | --- | --- | --- | --- | --- | --- |
|  | **CONTROL (SD)** | **PATIENTS (SD)** | **p** | **CONTROL (SD)** | **PATIENTS (SD)** | **p** |
| 1000/2000 (40) |  |  |  |  |  |  |
| STD | 8.26E-04 (2.73E-05) | 8.78E-04 (4.46E-05) | <0.001 (0.70) | 2.15E-01 (5.92E-03) | 2.06E-01 (8.91E-03) | <0.001 (0.54) |
| BSD | 8.24E-04 (2.79E-05) | 8.76E-04 (4.45E-05) | <0.001 (0.66) | 2.15E-01 (6.05E-03) | 2.06E-01 (8.61E-03) | <0.001 (0.56) |
| p | <0.05 (0.48) | <0.01 (0.63) |  | 0.126 (0.34) | 0.332 (0.22) |  |
| 2000 (20) |  |  |  |  |  |  |
| STD | 7.66E-04 (2.66E-05) | 8.12E-04 (3.92E-05) | <0.001 (0.67) | 2.29E-01 (6.10E-03) | 2.20E-01 (7.85E-03) | <0.001 (0.56) |
| BSD | 7.65E-04 (2.72E-05) | 8.10E-04 (3.91E-05) | <0.001 (0.63) | 2.30E-01 (6.10E-03) | 2.21E-01 (7.61E-03) | <0.001 (0.57) |
| p | 0.126 (0.34) | <0.01 (0.61) |  | <0.005 (0.73) | 0.079 (0.39) |  |
| 1000 (20) |  |  |  |  |  |  |
| STD | 1.03E-03 (3.64E-05) | 1.11E-03 (6.75E-05) | <0.001 (0.68) | 2.39E-01 (6.81E-03) | 2.30E-01 (8.31E-03) | <0.001 (0.53) |
| BSD | 1.02E-03 (3.69E-05) | 1.10E-03 (6.70E-05) | <0.001 (0.68) | 2.40E-01 (7.06E-03) | 2.31E-01 (8.61E-03) | <0.005 (0.52) |
| p | <0.001 (0.88) | <0.001 (0.88) |  | <0.05 (0.52) | 0.191 (0.29) |  |
| 1000 (11) |  |  |  |  |  |  |
| STD | 1.02E-03 (3.83E-05) | 1.10E-03 (6.73E-05) | <0.001 (0.67) | 2.76E-01 (9.04E-03) | 2.67E-01 (8.93E-03) | <0.01 (0.43) |
| BSD | 1.01E-03 (3.88E-05) | 1.09E-03 (6.65E-05) | <0.001 (0.67) | 2.76E-01 (9.13E-03) | 2.68E-01 (9.36E-03) | <0.005 (0.45) |
| p | <0.001 (0.88) | <0.001 (0.88) |  | <0.005 (0.72) | <0.001 (0.83) |  |
| 1000 (6) |  |  |  |  |  |  |
| STD | 9.86E-04 (4.39E-05) | 1.06E-03 (6.84E-05) | <0.001 (0.62) | 3.12E-01 (8.06E-03) | 3.01E-01 (8.62E-03) | <0.001 (0.57) |
| BSD | 9.83E-04 (4.45E-05) | 1.06E-03 (6.86E-05) | <0.001 (0.62) | 3.12E-01 (8.20E-03) | 3.01E-01 (9.06E-03) | <0.001 (0.57) |
| p | <0.001 (0.82) | <0.01 (0.61) |  | <0.01 (0.58) | 0.601 (0.12) |  |

**30**

| B val (dirs) | Mean Diffusivity | | | Fractional Anisotropy | | |
| --- | --- | --- | --- | --- | --- | --- |
|  | **CONTROL (SD)** | **PATIENTS (SD)** | **p** | **CONTROL (SD)** | **PATIENTS (SD)** | **p** |
| 1000/2000 (40) |  |  |  |  |  |  |
| STD | 8.33E-04 (3.16E-05) | 8.84E-04 (5.00E-05) | <0.001 (0.59) | 2.15E-01 (6.84E-03) | 2.05E-01 (9.63E-03) | <0.001 (0.52) |
| BSD | 8.31E-04 (3.16E-05) | 8.82E-04 (5.04E-05) | <0.001 (0.57) | 2.16E-01 (6.93E-03) | 2.05E-01 (9.37E-03) | <0.001 (0.53) |
| p | <0.001 (0.64) | <0.001 (0.71) |  | 0.329 (0.18) | 0.171 (0.25) |  |
| 2000 (20) |  |  |  |  |  |  |
| STD | 7.72E-04 (2.91E-05) | 8.18E-04 (4.44E-05) | <0.001 (0.59) | 2.30E-01 (6.53E-03) | 2.20E-01 (8.86E-03) | <0.001 (0.57) |
| BSD | 7.71E-04 (2.92E-05) | 8.16E-04 (4.47E-05) | <0.001 (0.56) | 2.30E-01 (6.52E-03) | 2.20E-01 (8.62E-03) | <0.001 (0.59) |
| p | <0.005 (0.55) | <0.001 (0.69) |  | <0.001 (0.62) | <0.01 (0.48) |  |
| 1000 (20) |  |  |  |  |  |  |
| STD | 1.05E-03 (4.62E-05) | 1.12E-03 (7.46E-05) | <0.001 (0.56) | 2.39E-01 (7.06E-03) | 2.29E-01 (9.49E-03) | <0.001 (0.56) |
| BSD | 1.04E-03 (4.61E-05) | 1.11E-03 (7.42E-05) | <0.001 (0.56) | 2.40E-01 (7.20E-03) | 2.29E-01 (9.60E-03) | <0.001 (0.54) |
| p | <0.001 (0.87) | <0.001 (0.87) |  | 0.153 (0.26) | 0.072 (0.33) |  |
| 1000 (11) |  |  |  |  |  |  |
| STD | 1.03E-03 (4.73E-05) | 1.11E-03 (7.46E-05) | <0.001 (0.55) | 2.76E-01 (8.54E-03) | 2.64E-01 (1.06E-02) | <0.001 (0.52) |
| BSD | 1.02E-03 (4.73E-05) | 1.10E-03 (7.41E-05) | <0.001 (0.55) | 2.77E-01 (8.60E-03) | 2.65E-01 (1.08E-02) | <0.001 (0.53) |
| p | <0.001 (0.87) | <0.001 (0.87) |  | <0.005 (0.60) | <0.001 (0.79) |  |
| 1000 (6) |  |  |  |  |  |  |
| STD | 9.97E-04 (5.06E-05) | 1.07E-03 (7.57E-05) | <0.001 (0.53) | 3.13E-01 (8.45E-03) | 2.99E-01 (1.02E-02) | <0.001 (0.65) |
| BSD | 9.94E-04 (5.07E-05) | 1.07E-03 (7.63E-05) | <0.001 (0.53) | 3.13E-01 (8.49E-03) | 2.99E-01 (1.03E-02) | <0.001 (0.64) |
| p | <0.001 (0.84) | <0.001 (0.67) |  | 0.069 (0.33) | 0.797 (0.05) |  |

**40**

| B val (dirs) | Mean Diffusivity | | | Fractional Anisotropy | | |
| --- | --- | --- | --- | --- | --- | --- |
|  | **CONTROL (SD)** | **PATIENTS (SD)** | **p** | **CONTROL (SD)** | **PATIENTS (SD)** | **p** |
| 1000/2000 (40) |  |  |  |  |  |  |
| STD | 8.34E-04 (2.96E-05) | 8.85E-04 (4.86E-05) | <0.001 (0.58) | 2.15E-01 (7.46E-03) | 2.06E-01 (9.52E-03) | <0.001 (0.45) |
| BSD | 8.32E-04 (2.96E-05) | 8.82E-04 (4.87E-05) | <0.001 (0.56) | 2.15E-01 (7.48E-03) | 2.07E-01 (9.30E-03) | <0.001 (0.46) |
| p | <0.001 (0.73) | <0.001 (0.74) |  | 0.354 (0.15) | 0.101 (0.26) |  |
| 2000 (20) |  |  |  |  |  |  |
| STD | 7.73E-04 (2.72E-05) | 8.19E-04 (4.35E-05) | <0.001 (0.59) | 2.30E-01 (6.81E-03) | 2.20E-01 (8.82E-03) | <0.001 (0.52) |
| BSD | 7.71E-04 (2.72E-05) | 8.16E-04 (4.36E-05) | <0.001 (0.58) | 2.30E-01 (6.76E-03) | 2.21E-01 (8.61E-03) | <0.001 (0.54) |
| p | <0.001 (0.66) | <0.001 (0.71) |  | <0.001 (0.57) | <0.001 (0.54) |  |
| 1000 (20) |  |  |  |  |  |  |
| STD | 1.05E-03 (4.43E-05) | 1.12E-03 (7.15E-05) | <0.001 (0.52) | 2.39E-01 (7.37E-03) | 2.29E-01 (9.68E-03) | <0.001 (0.52) |
| BSD | 1.04E-03 (4.42E-05) | 1.11E-03 (7.13E-05) | <0.001 (0.52) | 2.40E-01 (7.43E-03) | 2.30E-01 (9.82E-03) | <0.001 (0.50) |
| p | <0.001 (0.87) | <0.001 (0.87) |  | 0.460 (0.12) | 0.083 (0.27) |  |
| 1000 (11) |  |  |  |  |  |  |
| STD | 1.04E-03 (4.50E-05) | 1.11E-03 (7.17E-05) | <0.001 (0.52) | 2.77E-01 (8.76E-03) | 2.64E-01 (1.10E-02) | <0.001 (0.54) |
| BSD | 1.03E-03 (4.50E-05) | 1.10E-03 (7.13E-05) | <0.001 (0.52) | 2.77E-01 (8.74E-03) | 2.65E-01 (1.12E-02) | <0.001 (0.54) |
| p | <0.001 (0.87) | <0.001 (0.87) |  | <0.001 (0.56) | <0.001 (0.73) |  |
| 1000 (6) |  |  |  |  |  |  |
| STD | 1.00E-03 (4.81E-05) | 1.07E-03 (7.34E-05) | <0.001 (0.51) | 3.14E-01 (8.42E-03) | 2.99E-01 (1.06E-02) | <0.001 (0.64) |
| BSD | 9.97E-04 (4.81E-05) | 1.07E-03 (7.40E-05) | <0.001 (0.51) | 3.14E-01 (8.39E-03) | 2.99E-01 (1.08E-02) | <0.001 (0.64) |
| p | <0.001 (0.85) | <0.001 (0.74) |  | 0.727 (0.06) | 0.904 (0.02) |  |

**50**

| B val (dirs) | Mean Diffusivity | | | Fractional Anisotropy | | |
| --- | --- | --- | --- | --- | --- | --- |
|  | **CONTROL (SD)** | **PATIENTS (SD)** | **p** | **CONTROL (SD)** | **PATIENTS (SD)** | **p** |
| 1000/2000 (40) |  |  |  |  |  |  |
| STD | 8.33E-04 (3.16E-05) | 8.79E-04 (4.83E-05) | <0.001 (0.51) | 2.15E-01 (7.97E-03) | 2.07E-01 (9.97E-03) | <0.001 (0.41) |
| BSD | 8.30E-04 (3.17E-05) | 8.77E-04 (4.86E-05) | <0.001 (0.50) | 2.15E-01 (7.92E-03) | 2.07E-01 (9.73E-03) | <0.001 (0.42) |
| p | <0.001 (0.77) | <0.001 (0.69) |  | 0.490 (0.10) | 0.157 (0.20) |  |
| 2000 (20) |  |  |  |  |  |  |
| STD | 7.71E-04 (2.88E-05) | 8.14E-04 (4.32E-05) | <0.001 (0.52) | 2.30E-01 (7.45E-03) | 2.21E-01 (9.11E-03) | <0.001 (0.47) |
| BSD | 7.69E-04 (2.90E-05) | 8.12E-04 (4.35E-05) | <0.001 (0.52) | 2.30E-01 (7.32E-03) | 2.21E-01 (8.86E-03) | <0.001 (0.49) |
| p | <0.001 (0.71) | <0.001 (0.64) |  | <0.001 (0.51) | <0.001 (0.51) |  |
| 1000 (20) |  |  |  |  |  |  |
| STD | 1.05E-03 (4.75E-05) | 1.11E-03 (7.11E-05) | <0.001 (0.45) | 2.39E-01 (8.25E-03) | 2.30E-01 (9.85E-03) | <0.001 (0.46) |
| BSD | 1.04E-03 (4.75E-05) | 1.10E-03 (7.11E-05) | <0.001 (0.45) | 2.40E-01 (8.23E-03) | 2.30E-01 (9.88E-03) | <0.001 (0.45) |
| p | <0.001 (0.87) | <0.001 (0.87) |  | 0.595 (0.08) | 0.052 (0.28) |  |
| 1000 (11) |  |  |  |  |  |  |
| STD | 1.04E-03 (4.81E-05) | 1.10E-03 (7.13E-05) | <0.001 (0.46) | 2.77E-01 (9.87E-03) | 2.65E-01 (1.11E-02) | <0.001 (0.50) |
| BSD | 1.03E-03 (4.81E-05) | 1.09E-03 (7.11E-05) | <0.001 (0.45) | 2.78E-01 (9.76E-03) | 2.65E-01 (1.12E-02) | <0.001 (0.51) |
| p | <0.001 (0.87) | <0.001 (0.87) |  | <0.001 (0.55) | <0.001 (0.68) |  |
| 1000 (6) |  |  |  |  |  |  |
| STD | 9.98E-04 (5.11E-05) | 1.06E-03 (7.28E-05) | <0.001 (0.44) | 3.14E-01 (9.51E-03) | 2.99E-01 (1.08E-02) | <0.001 (0.63) |
| BSD | 9.95E-04 (5.10E-05) | 1.06E-03 (7.34E-05) | <0.001 (0.44) | 3.14E-01 (9.42E-03) | 2.99E-01 (1.09E-02) | <0.001 (0.62) |
| p | <0.001 (0.85) | <0.001 (0.77) |  | 0.904 (0.02) | 0.640 (0.07) |  |

Note. – Data are mean with standard deviation (SD) in parentheses.

STD = standard, BSD = B-matrix Spatial Distribution. Mean diffusivity is given in mm^2^/s. A p-value less than .05 indicates a statistically significant difference; the corresponding effect size is specified in parentheses alongside.

**Table S2a**. Diffusion tensor metrics from 50 healthy control measurements (CONTROL) and 50 multiple sclerosis patients (PATIENTS) in standard (STD) approach and after eliminating systematic errors (BSD) for White Matter ROI.

| B val (dirs) | Mean Diffusivity | | | Fractional Anisotropy | | |
| --- | --- | --- | --- | --- | --- | --- |
|  | **CONTROL (SD)** | **PATIENTS (SD)** | **p** | **CONTROL (SD)** | **PATIENTS (SD)** | **p** |
| 1000/2000 (40) |  |  |  |  |  |  |
| STD | 6.84E-04 (1.59E-05) | 7.06E-04 (2.31E-05) | <0.001 (0.48) | 3.56E-01 (1.50E-02) | 3.51E-01 (1.65E-02) | 0.189 (0.13) |
| BSD | 6.77E-04 (1.56E-05) | 6.99E-04 (2.28E-05) | <0.001 (0.49) | 3.55E-01 (1.51E-02) | 3.50E-01 (1.64E-02) | 0.203 (0.13) |
| p | <0.001 (0.87) | <0.001 (0.87) |  | <0.001 (0.82) | <0.001 (0.87) |  |
| 2000 (20) |  |  |  |  |  |  |
| STD | 6.58E-04 (1.56E-05) | 6.79E-04 (2.24E-05) | <0.001 (0.49) | 3.57E-01 (1.46E-02) | 3.51E-01 (1.64E-02) | 0.159 (0.14) |
| BSD | 6.51E-04 (1.54E-05) | 6.73E-04 (2.22E-05) | <0.001 (0.49) | 3.56E-01 (1.47E-02) | 3.50E-01 (1.62E-02) | 0.159 (0.14) |
| p | <0.001 (0.87) | <0.001 (0.87) |  | <0.001 (0.80) | <0.001 (0.87) |  |
| 1000 (20) |  |  |  |  |  |  |
| STD | 7.89E-04 (1.78E-05) | 8.11E-04 (2.60E-05) | <0.001 (0.47) | 3.68E-01 (1.58E-02) | 3.61E-01 (1.70E-02) | 0.104 (0.17) |
| BSD | 7.81E-04 (1.73E-05) | 8.03E-04 (2.55E-05) | <0.001 (0.47) | 3.66E-01 (1.58E-02) | 3.59E-01 (1.70E-02) | 0.108 (0.16) |
| p | <0.001 (0.87) | <0.001 (0.87) |  | <0.001 (0.84) | <0.001 (0.86) |  |
| 1000 (11) |  |  |  |  |  |  |
| STD | 7.89E-04 (1.82E-05) | 8.13E-04 (2.63E-05) | <0.001 (0.47) | 3.80E-01 (1.54E-02) | 3.70E-01 (1.73E-02) | <0.05 (0.25) |
| BSD | 7.81E-04 (1.79E-05) | 8.04E-04 (2.58E-05) | <0.001 (0.48) | 3.79E-01 (1.54E-02) | 3.69E-01 (1.73E-02) | <0.01 (0.27) |
| p | <0.001 (0.87) | <0.001 (0.87) |  | <0.001 (0.82) | <0.001 (0.87) |  |
| 1000 (6) |  |  |  |  |  |  |
| STD | 7.77E-04 (1.95E-05) | 8.02E-04 (2.65E-05) | <0.001 (0.49) | 4.11E-01 (1.40E-02) | 3.99E-01 (1.70E-02) | <0.005 (0.34) |
| BSD | 7.72E-04 (1.87E-05) | 7.97E-04 (2.63E-05) | <0.001 (0.49) | 4.09E-01 (1.41E-02) | 3.97E-01 (1.71E-02) | <0.001 (0.35) |
| p | <0.001 (0.87) | <0.001 (0.83) |  | <0.001 (0.86) | <0.001 (0.87) |  |

Note. – Data are mean with standard deviation (SD) in parentheses.

STD = standard, BSD = B-matrix Spatial Distribution. Mean diffusivity is given in mm^2^/s. A p-value less than .05 indicates a statistically significant difference; the corresponding effect size is specified in parentheses alongside.

**Table S2b**. Diffusion tensor metrics from 50 healthy control measurements (CONTROL) and 50 multiple sclerosis patients (PATIENTS) in standard (STD) approach and after eliminating systematic errors (BSD) for Gray Matter ROI.

| B val (dirs) | Mean Diffusivity | | | Fractional Anisotropy | | |
| --- | --- | --- | --- | --- | --- | --- |
|  | **CONTROL (SD)** | **PATIENTS (SD)** | **p** | **CONTROL (SD)** | **PATIENTS (SD)** | **p** |
| 1000/2000 (40) |  |  |  |  |  |  |
| STD | 8.30E-04 (1.68E-05) | 8.48E-04 (2.46E-05) | <0.001 (0.39) | 1.37E-01 (4.25E-03) | 1.36E-01 (5.74E-03) | 0.143 (0.15) |
| BSD | 8.23E-04 (1.64E-05) | 8.41E-04 (2.48E-05) | <0.001 (0.39) | 1.37E-01 (4.24E-03) | 1.36E-01 (5.69E-03) | 0.104 (0.17) |
| p | <0.001 (0.87) | <0.001 (0.86) |  | 0.803 (0.04) | 0.593 (0.09) |  |
| 2000 (20) |  |  |  |  |  |  |
| STD | 7.97E-04 (1.50E-05) | 8.14E-04 (2.16E-05) | <0.001 (0.42) | 1.44E-01 (4.67E-03) | 1.42E-01 (6.18E-03) | <0.05 (0.22) |
| BSD | 7.91E-04 (1.45E-05) | 8.08E-04 (2.18E-05) | <0.001 (0.43) | 1.44E-01 (4.63E-03) | 1.42E-01 (6.15E-03) | <0.05 (0.24) |
| p | <0.001 (0.87) | <0.001 (0.86) |  | 0.220 (0.19) | 0.619 (0.08) |  |
| 1000 (20) |  |  |  |  |  |  |
| STD | 9.59E-04 (2.59E-05) | 9.80E-04 (3.73E-05) | <0.005 (0.31) | 1.44E-01 (5.64E-03) | 1.40E-01 (6.34E-03) | <0.005 (0.32) |
| BSD | 9.49E-04 (2.60E-05) | 9.71E-04 (3.73E-05) | <0.005 (0.31) | 1.43E-01 (5.70E-03) | 1.40E-01 (6.63E-03) | <0.005 (0.32) |
| p | <0.001 (0.87) | <0.001 (0.87) |  | 0.252 (0.18) | 0.619 (0.08) |  |
| 1000 (11) |  |  |  |  |  |  |
| STD | 9.59E-04 (2.61E-05) | 9.81E-04 (3.74E-05) | <0.005 (0.32) | 1.69E-01 (8.78E-03) | 1.61E-01 (9.19E-03) | <0.001 (0.50) |
| BSD | 9.48E-04 (2.63E-05) | 9.71E-04 (3.73E-05) | <0.005 (0.32) | 1.70E-01 (8.78E-03) | 1.61E-01 (9.56E-03) | <0.001 (0.51) |
| p | <0.001 (0.87) | <0.001 (0.87) |  | 0.360 (0.14) | 0.449 (0.12) |  |
| 1000 (6) |  |  |  |  |  |  |
| STD | 9.54E-04 (2.61E-05) | 9.75E-04 (3.74E-05) | <0.005 (0.31) | 2.23E-01 (1.38E-02) | 2.07E-01 (1.35E-02) | <0.001 (0.59) |
| BSD | 9.49E-04 (2.58E-05) | 9.70E-04 (3.80E-05) | <0.005 (0.30) | 2.23E-01 (1.37E-02) | 2.06E-01 (1.37E-02) | <0.001 (0.59) |
| p | <0.001 (0.87) | <0.001 (0.81) |  | <0.001 (0.49) | <0.001 (0.50) |  |

Note. – Data are mean with standard deviation (SD) in parentheses.

STD = standard, BSD = B-matrix Spatial Distribution. Mean diffusivity is given in mm^2^/s. A p-value less than .05 indicates a statistically significant difference; the corresponding effect size is specified in parentheses alongside.

**Figures**


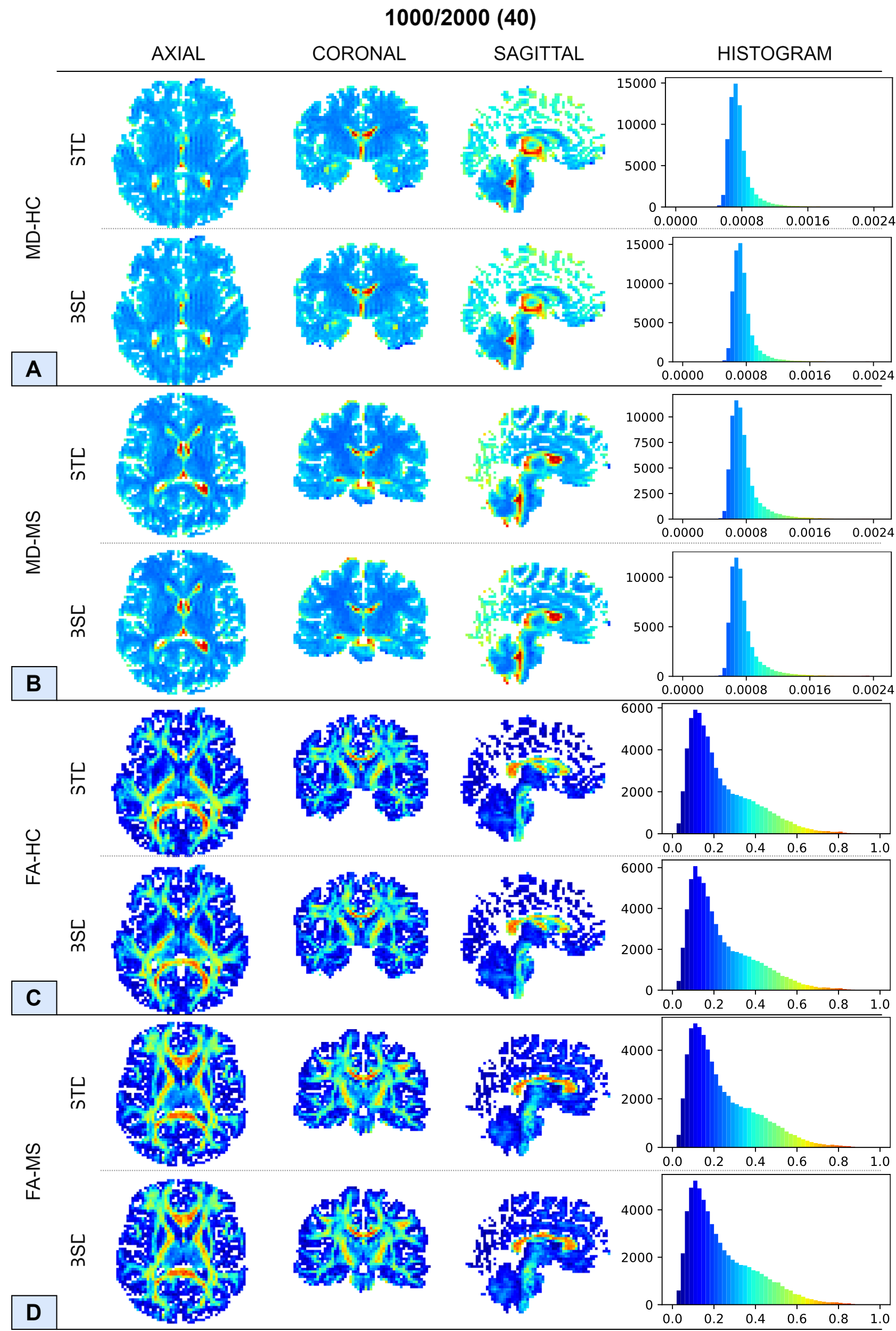


**Figure S1.** Spatial distribution of MD and FA applying the primary protocol 1000/2000(40) within the Whole Brain ROI in the standard approach (STD) and after eliminating systematic errors (BSD) for healthy controls (HC) and multiple sclerosis patients (MS).

STD = standard, BSD = B-matrix Spatial Distribution.

**
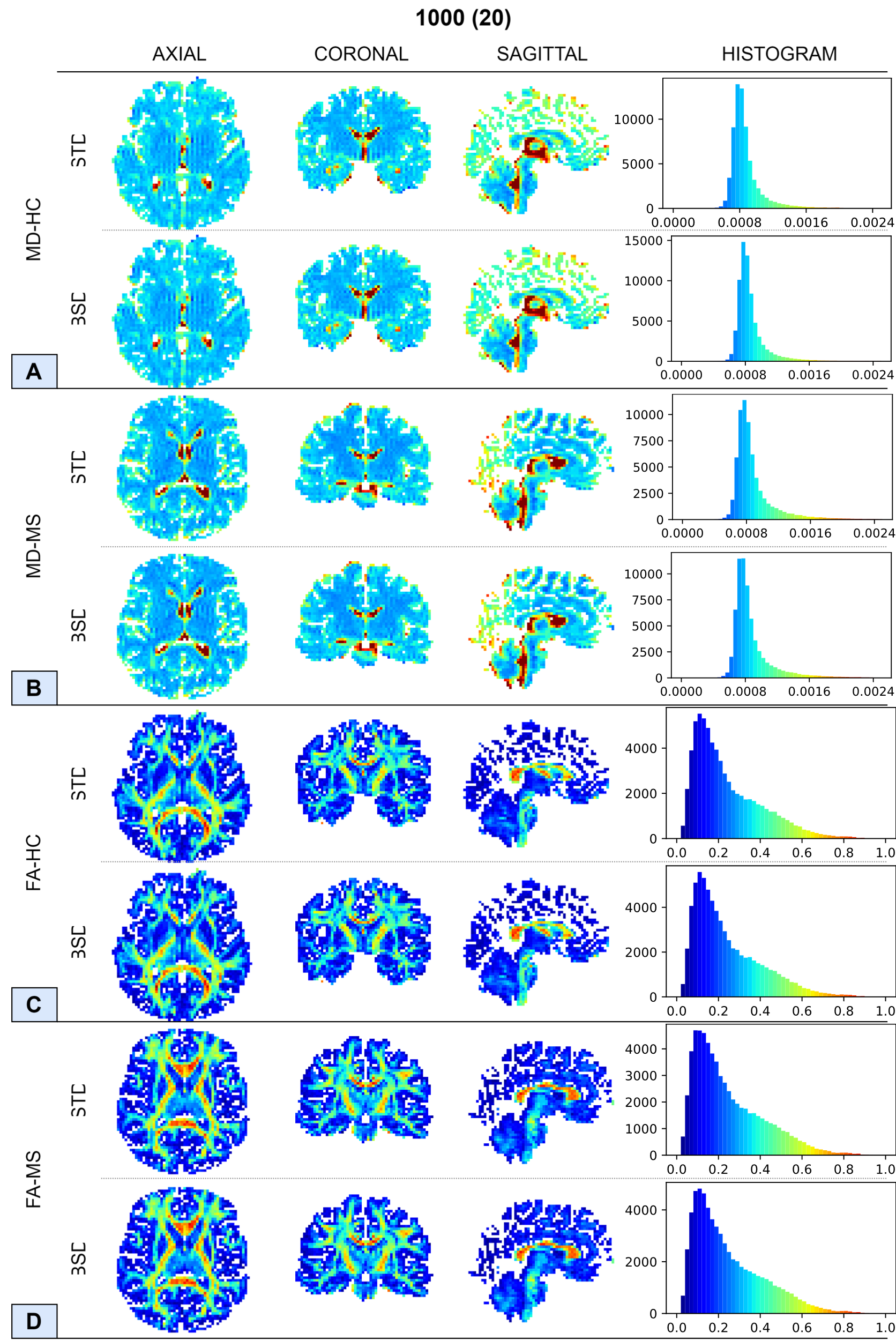
**

**Figure S2.** Spatial distribution of MD and FA applying the subset 1000(20) within the Whole Brain ROI in the standard approach (STD) and after eliminating systematic errors (BSD) for healthy controls (HC) and multiple sclerosis patients (MS).

STD = standard, BSD = B-matrix Spatial Distribution.


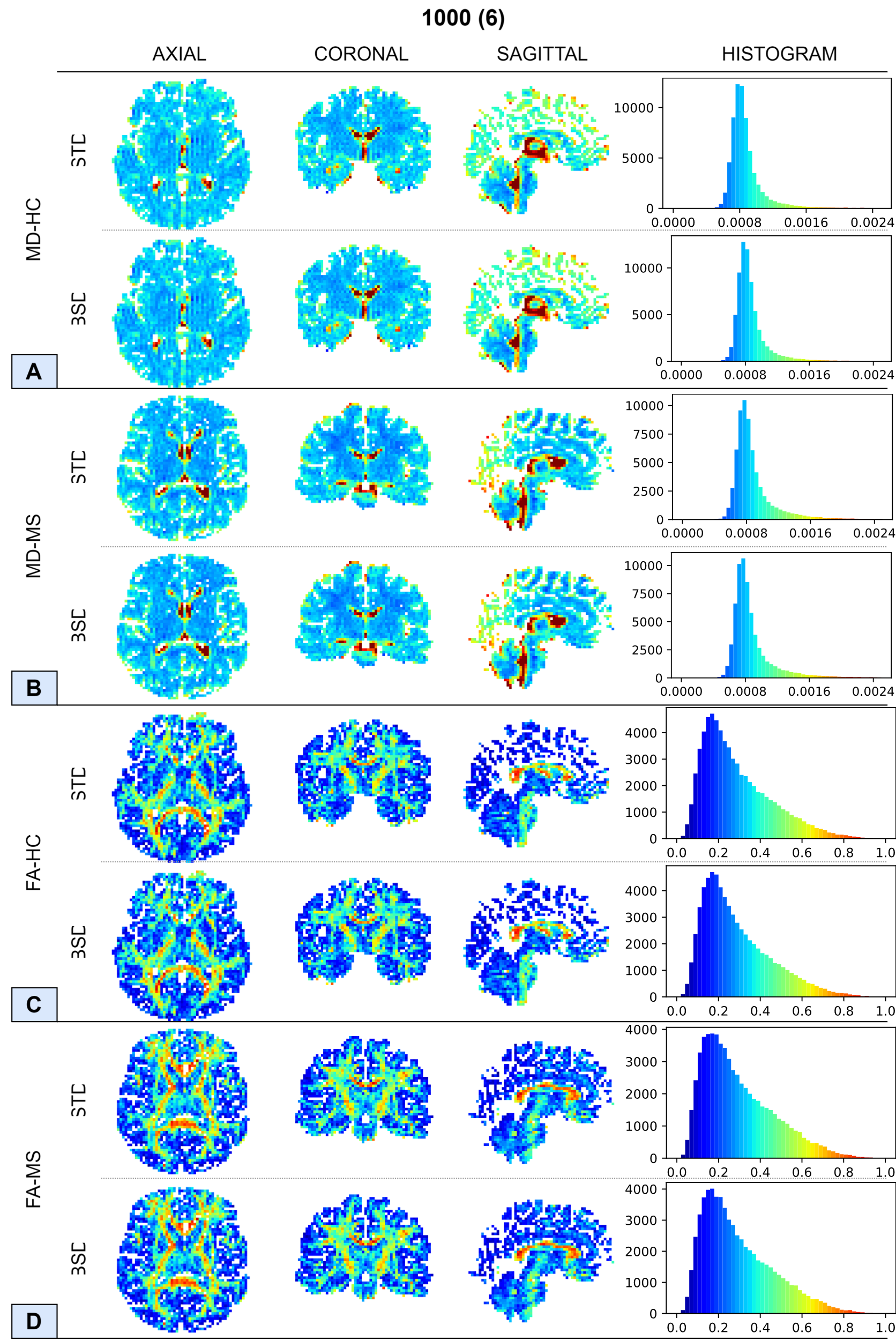


**Figure S3.** Spatial distribution of MD and FA applying the subset 1000(6) within the Whole Brain ROI in the standard approach (STD) and after eliminating systematic errors (BSD) for healthy controls (HC) and multiple sclerosis patients (MS).

STD = standard, BSD = B-matrix Spatial Distribution.

**Software and hardware specifications for reproducibility.**

All data processing and analyses were performed on a dedicated workstation with the following configuration:

Operating system & Development environment

- Microsoft Windows Server (version 2022)
- Microsoft Visual Studio 13.00 (C/C++ compiler; Microsoft, USA)

Software packages and versions:

- Statistical analysis: SciPy 1.12.0 (Python 3.11.9)
- Brain segmentation: FastSurfer
- DTI metrics & BSD-DTI: In-house software written in C/C++, compiled under Visual Studio 13.00. The algorithm usage is available at the link: https://nmrlab.pl/en/bsd/

Hardware:

- CPU: Dual Intel® Xeon® Gold 5220R processors (2.20 GHz base clock)
- GPU: 2 × NVIDIA RTX 3090 graphics cards
